# Knowledge, Attitude, and Practice Regarding Preconception Care: A Cross-Sectional Study Among Healthcare Workers in a Rural Block of Jammu and Kashmir, North India

**DOI:** 10.7759/cureus.109073

**Published:** 2026-05-18

**Authors:** Renu Sharma, Nitesh Kumar, Meeta Gupta, Harleen, Natasha Gupta

**Affiliations:** 1 Obstetrics and Gynaecology, All India Institute of Medical Sciences, Vijaypur, Jammu, IND; 2 Community Medicine, All India Institute of Medical Sciences, Vijaypur, Jammu, IND

**Keywords:** attitude, healthcare workers, knowledge, practice, preconception care

## Abstract

Background

Preconception care (PCC) improves maternal and child health outcomes by optimizing women's and couples' health before pregnancy through health education, supplementary medication, dietary modifications, and reducing preconception risk factors. In 2013, the World Health Organization (WHO) directed all nations to develop their own PCC policies based on national needs. The government of India included PCC as a key strategy to reduce perinatal mortality and morbidity in the India Newborn Action Plan (INAP) in 2014. However, its implementation remains limited in India. Healthcare workers, who provide primary health care in rural areas, are frontline personnel capable of playing a vital role in delivering PCC in these regions. This study was conducted with the aim of assessing the level of knowledge, attitude, and practice regarding PCC among healthcare workers in a rural block of Jammu and Kashmir, North India.

Methods

This cross-sectional study was conducted from July 2024 to July 2025. A semi-structured questionnaire was used to assess their knowledge, attitude, and practice concerning PCC. Data were described using frequencies, proportions, means, and standard deviations. The chi-square test was employed to analyze associations in bivariate analysis.

Results

Among 115 healthcare workers, 58 (50.4%) were Accredited Social Health Activist (ASHA) workers, 38 (33.1%) were Auxiliary Nurse Midwives (ANMs), General Nursing and Midwifery (GNMs), Multipurpose Health Workers (MPHWs), or Lady Health Visitors (LHVs), and 19 (16.5%) were medical officers. Of the participants, 68 (59.1%) had poor knowledge, and 66 (57.4%) displayed a negative attitude. Only 21 (18.3%) of them demonstrated good practices. Gaps were observed in understanding various PCC components, including preconception folic acid dosage, the adverse effects of obesity, intimate partner violence, exposure to environmental toxins, and the teratogenic effects of drugs. The level of education and professional background were significantly associated with knowledge, practice, and attitude scores.

Conclusions

Rural healthcare workers in India exhibit limited knowledge, negative attitudes, and inadequate practices regarding PCC. Training programs may help improve their knowledge, foster a positive attitude, and enhance the practice of PCC, thereby bridging the gap in the continuum of care.

## Introduction

Preconception care (PCC) focuses on health interventions for women and couples of reproductive age before conception, aiming to enhance their overall health and subsequently improve pregnancy and child health outcomes [1].

Risk factors, including nutritional problems (anemia, undernutrition, obesity), behavioral issues (smoking, alcoholism), social challenges (teenage pregnancies), and chronic medical conditions (diabetes, hypertension), negatively impact the pregnancy outcomes and increase the morbidity and mortality of mothers and children [2-7].

As per the National Family Health Survey-5 (NFHS-5), 2019-21, of India, 57% of adult women are anemic, 18.7% have low BMI, and 24% are obese. Moreover, 23.3% of women were married before the age of 18 years, and 6.8% were pregnant before 19 years [8]. Consequently, many are not in their best of health at the time of conception. The data show that half of the pregnancies are unplanned [9]. In our country, barely one-third of the women receive timely, sufficient, and appropriate antenatal care by skilled health personnel [10]. By the time a pregnant woman seeks her first antenatal consultation, preconception risk factors have already affected the growing embryo during the period of organogenesis (six to nine weeks). Thus, interventions initiated before pregnancy in the form of preconception health education, lifestyle modifications, supplementary medications, and screening and management of risk factors improve pregnancy outcomes [11].

In 2013, the World Health Organization (WHO) created a PCC package and emphasized the necessity of national policies and programs for the implementation of PCC. PCC was incorporated in the India Newborn Action Plan (INAP) in 2014 as one of its components to decrease perinatal mortality and morbidity. Despite this, PCC is undermined in India. A study conducted in rural Maharashtra shows that a mere 14.5% of women receive a health consultation prior to pregnancy [9]. The primary focus is on adolescent health, antenatal care, and child health within the framework of the Reproductive Maternal, Neonatal, Child and Adolescent Health (RMNCH + A) strategy of the National Health Mission, while PCC remains neglected, resulting in a discontinuity of care [12]. Healthcare workers in the public sector are pivotal in executing health programs in rural India. To provide PCC to this population, the role of healthcare workers is essential. Global studies indicate a significant deficiency in knowledge regarding PCC among healthcare workers [13-17]. There is a dearth of scientific evidence from our country about the knowledge, attitude, and practice (KAP) of PCC among healthcare providers.

This study was conducted with the aim of assessing the level of KAP regarding PCC among healthcare workers in a rural block of Jammu and Kashmir, North India.

## Materials and methods

Study setting and design

This cross-sectional study was conducted among healthcare workers providing services in public health facilities in the rural Block Ramgarh, located in District Samba, Jammu and Kashmir, India, from July 2024 to July 2025. These health facilities include one Community Health Center (CHC), two Primary Health Centers (PHCs), and 14 Health and Wellness Centers (HWCs).

Study population

The study population comprised all the healthcare workers of these public health facilities of Block Ramgarh. These health workers included Accredited Social Health Activist (ASHA) workers, Lady Health Visitors (LHVs), Auxiliary Nurse Midwives (ANMs), Mid-Level Health Providers (MLHPs), and medical officers. Those workers who had been employed for less than 6 months and those who did not provide consent were excluded from the study.

Sample size

The sample size was calculated using the formula n = (Z α/2)² P(1-P)/d², assuming a frequency of knowledge (P) as 31% with a 95% confidence level and a 5% absolute precision [13]. The initial sample size was 329. However, the total number of healthcare workers in Block Ramgarh was limited (N = 126). Hence, the finite population correction factor was applied with a 10% non-response rate, and the final sample size came out to be 103.

The study used an attempted census approach to include all eligible healthcare workers. A list of all 126 healthcare providers of the study block was obtained from the concerned authority, which was the Block Medical Officer (BMO), Ramgarh. Out of these 126 healthcare workers, seven were ineligible, as they had been employed for less than six months. Two of them did not give consent to participate, and two had not properly completed their questionnaires. Consequently, 115 healthcare workers who were eligible, gave their consent, and had duly completed their questionnaires were included in the study.

Data collection

After permission from local health authorities, health workers were mobilized to the nearest health facility, PHC Nandpur or CHC Ramgarh, with the help of facilitators on multiple occasions. Data were collected using a semi-structured KAP questionnaire (Appendices) on PCC. The data collectors and supervisors were given two days of training on the study materials and data collection procedures to ensure the quality of the study. Furthermore, supervisors and investigators reviewed and checked the completeness and consistency of the collected data.

Study tool and validation

This semi-structured KAP questionnaire on PCC was developed in English following a review of relevant literature [13,14]. The questionnaire was comprehensively designed, and a panel of specialists in obstetrics and gynecology and community medicine evaluated it for relevance, clarity, and domain coverage to establish the content validity. On their suggestions, necessary changes were made. The questionnaire was then translated into Hindi and back-translated into English by an independent translator to ensure linguistic and conceptual equivalence. The questionnaire was pretested on 20 participants in another setting similar to the study population to assess feasibility, clarity, and comprehensibility. Minor refinements were done to improve clarity and flow based on pretesting. Negatively worded items were reverse-scored prior to analysis. However, internal consistency reliability using Cronbach’s alpha was not formally assessed, which may be considered a limitation of the study.

The KAP questionnaire comprised four sections. The first section addressed the socio-demographic attributes of healthcare providers. The second section focused on the knowledge component, with 15 questions, each with a single correct answer. Each correct answer received a score of 1, while each incorrect response was assigned a score of 0. The participants, who scored below the mean score, were categorized as healthcare workers with poor/low knowledge. The third section addressed their attitude. It was evaluated using seven items, each with five-point Likert scale responses with a minimum possible score of 0 and a maximum possible score of 28. A score of more than 50% was considered a good attitude. The fourth section, designated as the Practice section, consisted of 11 questions assessing the frequency of practice over the preceding six months. Each question had an option response measuring frequency of practice as never = 0, rarely = 1, sometimes = 2, often = 3, and always = 4. The minimum possible score was 0, and the maximum possible score was 44. A score of more than 50% was considered good PCC practice, and less than 50% poor practice. The use of mean-based scoring and a 50% criterion for categorization of KAP was influenced by its application in previous studies [13,14]. In the context of PCC - where adequate knowledge and appropriate practices are essential for optimizing maternal and neonatal outcomes - a score below 50% reflects insufficient preparedness for effective PCC.

Statistical analysis

The data entry and analysis were done using the Statistical Package for Social Sciences (SPSS) (IBM SPSS Statistics for Windows, IBM Corp., Version 25.0, Armonk, NY). It delineated the data as frequencies and proportions for qualitative variables and means and standard deviations for quantitative variables. A chi-square test of association was used for bivariate analysis. A p-value < 0.05 was considered statistically significant.

## Results

Sociodemographic characteristics

Out of the 115 healthcare workers included in the study, 110 (95.6%) were females. All participants were more than 30 years of age, with 74.8% over 40 years old. Fifty percent were ASHA workers; 33.1% were ANMs, GNMs, MPHWs, or LHVs with a healthcare diploma; and 16.5% were medical officers. Out of all, 79.1% had five years or more of work experience in the field. Most of the ASHA workers had schooling up to the secondary level. Those working at HWC, PHC, and CHC were 67%, 21.7%, and 11.3%, respectively (Table 1).

**Table 1:** Sociodemographic characteristics of rural healthcare workers of Jammu and Kashmir in North India (N = 115) ANM - Auxiliary Nurse Midwife; ASHA - Accredited Social Health Activist; CHC - Community Health Centre; GNM - General Nursing and Midwifery; HWC - Health and Wellness Centre; LHV - Lady Health Visitor; MPHW - Multipurpose Health Worker; PHC - Primary Health Centre

Sociodemographic Characteristics		Frequency (N)	Percentage (%)
Gender	Male	5	4.4
	Female	110	95.6
Age groups	21-30	0	0
	31-40	29	25.2
	>40	86	74.8
Religion	Hindu	99	86.1
	Muslim	5	4.4
	Sikh	11	9.5
	Christian	0	0
	Others	0	0
Marital status	Married	105	91.3
	Unmarried	4	3.5
	Divorced/separated	0	0
	Widow/widower	6	5.2
Education	Secondary	64	55.7
	Higher secondary	25	21.7
	Graduation	23	20
	Post-graduation and above	3	2.6
Profession	ASHA Worker	58	50.4
	ANM, GNM, MPHW, LHV	38	33.1
	Medical officer	19	16.5
Work experience	<5 years	24	20.9
	≥5 years	91	79.1
Practice setting	CHC	13	11.3
	PHC	25	21.7
	HWC	77	67

Knowledge about PCC

**Figure 1:**
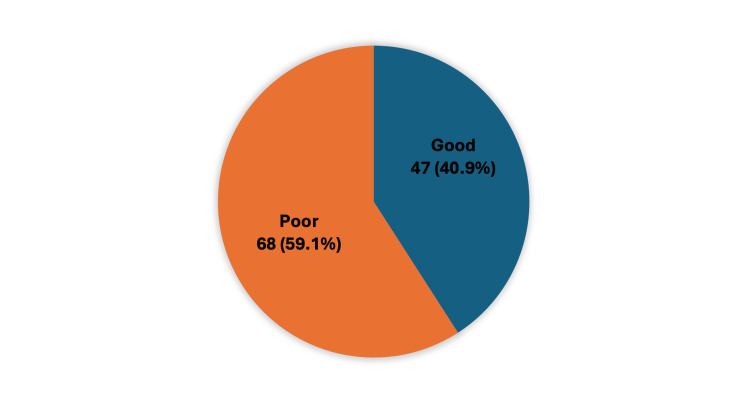
Knowledge of rural healthcare workers about preconception care (PCC) of Jammu and Kashmir in North India (N = 115) Good knowledge: N = 47 (40.9%); poor knowledge: N = 68 (59.1%).

As shown in Figure 1, out of 115 health workers, 68 (59.1%) had inadequate knowledge regarding PCC.

The mean knowledge score was 7.70 (SD ± 2.87). The lowest score recorded by any participant was zero. Knowledge related to different components of PCC was insufficient (Table 2).

**Table 2:** Knowledge of rural health care workers about preconception care (PCC) of Jammu and Kashmir in North India (N = 115)

S. No	Variables	Response Category	Frequency	Percentage
1	PCC is for adolescents and reproductive-age women.	True	105	91.3
		False	7	6.1
		Don’t know	3	2.6
2	PCC should be started 1month before conception in couples planning for pregnancy.	True	98	85.2
		False	11	9.6
		Don’t know	6	5.2
3	Folic acid should be started 3 months before pregnancy.	True	82	71.3
		False	24	20.9
		Don’t know	9	7.8
4	Anemia should be corrected only during pregnancy.	True	73	63.5
		False	36	31.3
		Don’t know	6	5.2
5	All women of reproductive age should take 0.4 mg (400 mcg) of folic acid daily.	True	42	36.5
		False	57	49.6
		Don’t know	16	13.9
6	Obesity does not lead to adverse pregnancy outcomes.	True	63	54.8
		False	44	38.2
		Don’t know	8	7.0
7	Exercise 30 minutes a day for at least five days is good for women planning for pregnancy.	True	80	59.6
		False	30	26.0
		Don’t know	5	4.4
8	Women who are taking anti-hypertensive before pregnancy should continue to take same medicines even during pregnancy.	True	85	73.9
		False	22	19.1
		Don’t know	8	7.0
9	Some antiepileptic drugs like phenytoin and valproate can lead to teratogenicity.	True	34	29.6
		False	62	53.9
		Don’t know	19	16.5
10	Rubella vaccine is contraindicated during pregnancy.	True	63	54.8
		False	38	33.0
		Don’t know	14	12.2
11	Intimate partner violence is not a concern for PCC.	True	57	49.6
		False	39	33.9
		Don’t know	19	16.5
12	Mental health is important for overall health of women and improves pregnancy outcomes.	True	106	92.2
		False	8	7.0
		Don’t know	1	0.8
13	Exposure to pesticides is a cause of concern for females planning for pregnancy.	True	27	23.5
		False	69	60.0
		Don’t know	19	16.5
14	Screening for sexually transmitted diseases should be done during PCC.	True	102	88.7
		False	5	4.3
		Don’t know	8	7.0
15	Family planning advice does not form a component of PCC.	True	71	61.7
		False	38	33.0
		Don’t know	6	5.3

The majority (91.3%) recognized that PCC is pertinent for all women of reproductive age, including adolescents. However, their understanding of the detrimental effects of obesity, the teratogenicity of drugs, such as antiepileptic drugs, and environmental toxins like pesticides was inadequate, with 61.8%, 70.4%, and 76.5%, respectively, lacking knowledge in these areas. While 82 of them (71.3%) were aware that folic acid should be initiated three months prior to conception, only 36.5% were informed about its appropriate dosage and schedule for folic acid. Additionally, 63.5% believed that the correction of anemia should be done only during the antenatal period. 

A small proportion of participants were aware of the necessity for drug modification, such as hypertension medications, before pregnancy (19.1%), and the impact of intimate partner violence (IPV) (33.9%). Additionally, 67% did not recognize family planning as an element of PCC.

However, they had substantial knowledge about some components, such as the importance of mental health, rubella vaccination, and screening of sexually transmitted diseases preconceptionally (92.2%, 54.8%, and 88.7%, respectively).

Attitude towards PCC

**Figure 2:**
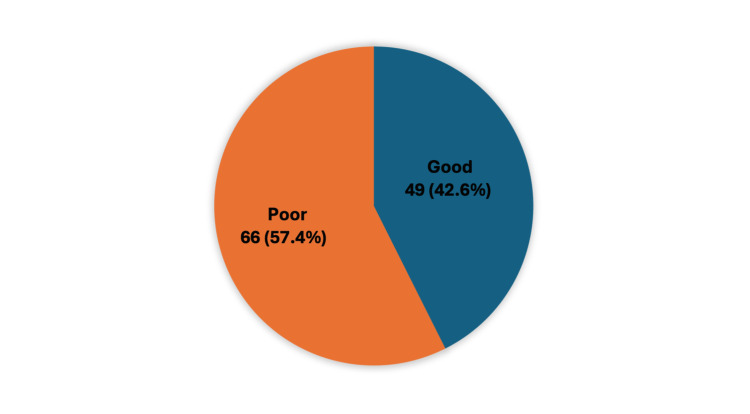
Attitude of rural healthcare workers about preconception care (PCC) of Jammu and Kashmir in North India (N = 115) Good attitude: N = 49 (42.6%); poor attitude: N = 66 (57.4%).

The attitude towards PCC was also poor, with 57.4% exhibiting a negative disposition (Figure 2).

While 64.4% concurred that PCC offers an opportunity to improve women's health prior to conception, a comparable 67% contended that PCC is less critical than antenatal care for pregnancy and child health outcomes. Additionally, 73% asserted that PCC is not essential for healthy couples. While 59.9% agreed that all healthcare professionals may readily incorporate the elements of PCC into their daily practice for all eligible individuals, 61.7% believe that PCC falls outside the scope of their services (Table 3).

**Table 3:** Attitude of rural healthcare workers about preconception care (PCC) of Jammu and Kashmir in North India (N = 115)

S. No.	Variable	Response Category	Frequency	Percentage
1	PCC provides an opportunity to optimize couples’ health particularly women’s health before conception.	Strongly disagree	36	31.3
		Disagree	5	4.3
		Undecided	0	0
		Agree	44	38.3
		Strongly agree	30	26.1
2	PCC services are not as important as antenatal services for better maternal and fetal outcomes.	Strongly disagree	14	12.2
		Disagree	21	18.2
		Undecided	3	2.6
		Agree	34	29.6
		Strongly agree	43	37.4
3	A hospital is not the best place to provide PCC.	Strongly disagree	57	49.6
		Disagree	29	25.2
		Undecided	6	5.2
		Agree	16	13.9
		Strongly agree	7	6.1
4	PCC is not required in individuals who are healthy.	Strongly disagree	6	5.2
		Disagree	19	16.6
		Undecided	6	5.2
		Agree	26	22.6
		Strongly agree	58	50.4
5	PCC is not within the scope of my professional responsibility.	Strongly disagree	21	18.3
		Disagree	17	14.8
		Undecided	6	5.2
		Agree	32	27.8
		Strongly agree	39	33.9
6	PCC should be given only to those suffering from critical diseases.	Strongly disagree	44	38.3
		Disagree	9	7.9
		Undecided	2	1.7
		Agree	38	33.0
		Strongly agree	22	19.1
7	All healthcare providers (professionals) can easily integrate the elements of PCC in their daily practice to all eligible individuals.	Strongly disagree	37	32.2
		Disagree	8	7.0
		Undecided	1	0.9
		Agree	44	38.2
		Strongly agree	25	21.7

Practice of PCC

**Figure 3:**
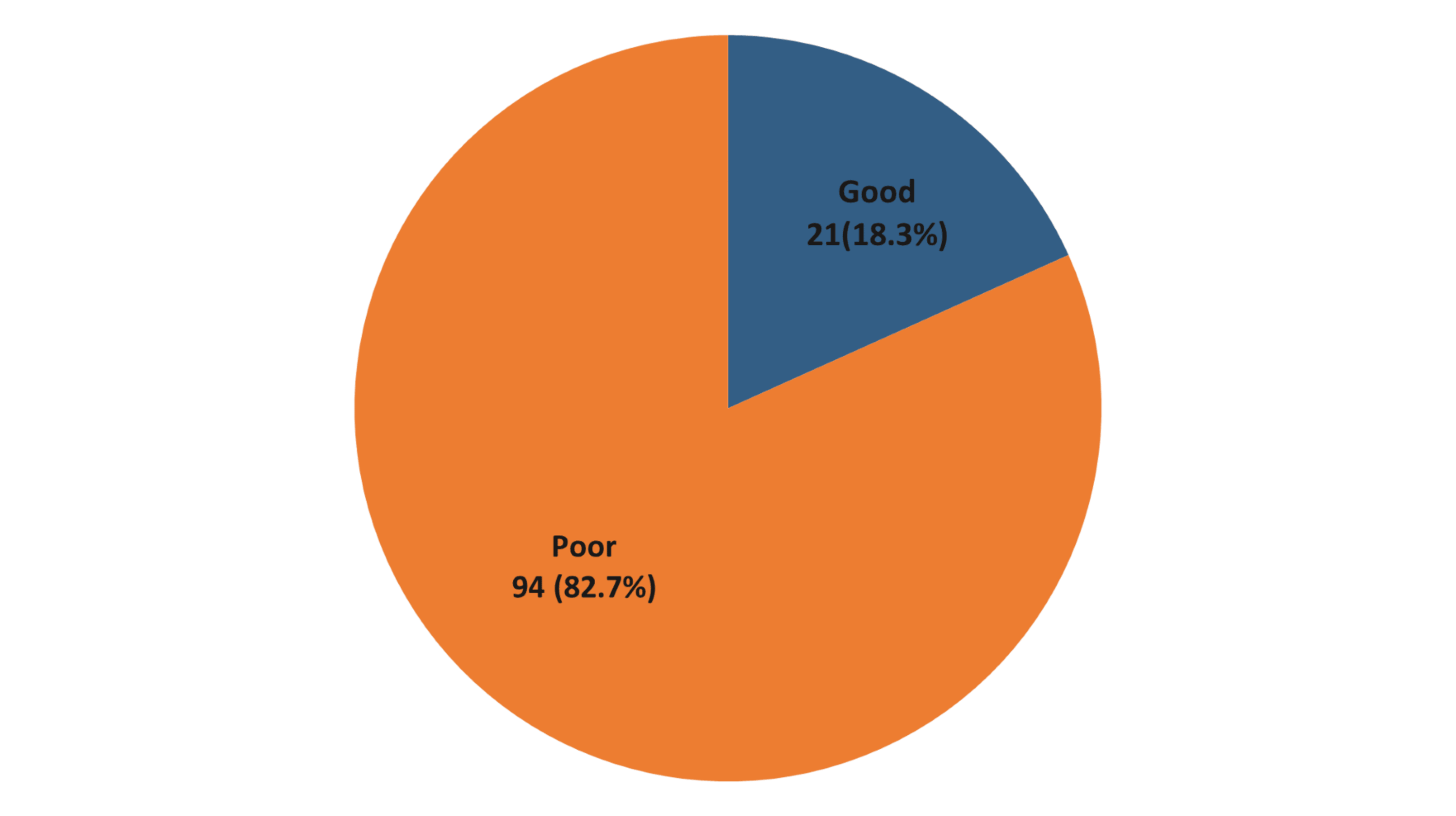
Practice of preconception care (PCC) by rural healthcare workers of Jammu and Kashmir in North India (N = 115) Good practice: N = 21 (18.3%); poor practice: N = 94 (82.7%).

Additionally, the practice of PCC was inadequate. Only 18.3% of them demonstrated good self-reported practice of PCC (Figure 3).

It was found that 84.3% of them never inquired about the family history or history of genetic diseases, which is crucial for identifying at-risk couples. The lifestyle behaviors, such as smoking and exercise, were consistently inquired about by only 14.8% of the participants. Likewise, only 24.3% offered dietary counseling. Merely, 10.4% of healthcare workers consistently recommended folic acid before conception. Certain components, such as ascertaining vaccination status and conducting nutritional assessments, were practiced effectively (81.8% and 53.0%, respectively). Appropriate referral to a higher center was being done by 74.8% of them (Table 4).

**Table 4:** Practice of preconception care (PCC) by rural health care workers PCC of Jammu and Kashmir in North India (N = 115)

S. No.	Variable	Response Category	Frequency	Percentage
1	Ask for past medical history	Never	44	38.3
		Rarely	17	14.8
		Sometimes	16	13.9
		Often	22	19.1
		Always	16	13.9
2	Ask for genetic history or family pedigree	Never	97	84.3
		Rarely	0	0
		Sometimes	0	0
		Often	10	8.7
		Always	8	7
3	Ask for lifestyle behaviors such as smoking, exercise, etc.	Never	43	37.3
		Rarely	16	14.0
		Sometimes	3	2.6
		Often	36	31.3
		Always	17	14.8
4	Ask for exposure to environmental toxins (agricultural/industrial)	Never	67	58.3
		Rarely	20	17.4
		Sometimes	7	6.1
		Often	11	9.6
		Always	10	8.6
5	Ask for drug history	Never	65	56.5
		Rarely	11	9.6
		Sometimes	8	6.9
		Often	11	9.6
		Always	20	17.4
6	Do nutritional assessment particularly body mass index (BMI)	Never	17	14.8
		Rarely	2	1.7
		Sometimes	35	30.5
		Often	33	28.7
		Always	28	24.3
7	Record the vaccination status	Never	16	13.9
		Rarely	0	0
		Sometimes	0	0
		Often	5	4.3
		Always	94	81.8
8	Do dietary counselling	Never	19	16.5
		Rarely	32	27.9
		Sometimes	25	21.7
		Often	11	9.6
		Always	28	24.3
9	Counselling for weight management	Never	49	42.6
		Rarely	24	20.9
		Sometimes	2	1.7
		Often	10	8.7
		Always	30	26.1
10	Prescribe preconception folic acid	Never	72	62.6
		Rarely	6	5.2
		Sometimes	5	4.4
		Often	20	17.4
		Always	12	10.4
11	Referral to higher center	Never	17	14.8
		Rarely	3	2.6
		Sometimes	3	2.6
		Often	6	5.2
		Always	86	74.8

KAP of healthcare workers was found to have a statistically significant association with their education level. The higher education level was associated with increased odds of adequate knowledge (OR 10.18, p-value < 0.001), positive attitude (OR 6.89, p-value < 0.001), and good practice (OR 7.2, p-value 0.03). Similarly, KAP regarding PCC was significantly associated with their professional background. ASHA workers were observed to have comparatively lower KAP scores (Table 5).

**Table 5:** Factors associated with knowledge, attitude, and practice of preconception care (PCC) among rural health care workers of Jammu and Kashmir in North India (N = 115) ANM - Auxiliary Nurse Midwife; CHC - Community Health Centre; GNM - General Nursing and Midwifery; HWC - Health and Wellness Centre; LHV - Lady Health Visitor; MPHW - Multipurpose Health Worker; PHC - Primary Health Centre

Variable	Knowledge			p-value	Attitude			p-value	Practice			p-value
	Poor	Good	Chi-square		Poor	Good	Chi-square		Good	Poor	Chi-square	
Gender												
Male	1	4	3.31 (OR 0.16)	0.069	1	4	2.99 (OR 0.17)	0.084	1	5	1.17 (OR 0.38)	0.285
Female	67	43			65	45			20	89		
Education												
≤Higher secondary	63	26	22.13 (OR 10.18)	<0.001	60	29	16.18 (OR 6.89)	<0.001	20	69	4.68 (OR 7.2)	0.030
≥Graduate	5	21			6	20			1	25		
Profession												
ASHA worker	51	7	47.27	<0.001	46	12	23.88	<0.001	17	41	9.63	0.008
ANM, GNM, MPHW, LHV	16	22			15	23			3	35		
Medical officer	1	18			5	14			1	18		
Work experience												
<5 years	12	12	1.05 (OR 0.62)	0.306	11	13	1.66 (OR 0.55)	0.198	2	22	2.00 (OR 0.34)	0.157
≥5 years	56	35			55	36			19	72		
Practice setting												
CHC	8	5	2.41	0.299	10	3	8.31	0.160	4	9	6.75	0.034
PHC	18	7			19	6			8	17		
HWC	42	35			37	40			9	68		

## Discussion

PCC is critical in the continuum of care to optimize the health of women and couples and prevent adverse pregnancy outcomes. PCC can be provided to the population at the grassroots level by healthcare workers functioning in these areas. The present study evaluated the knowledge, attitudes, and practices of these healthcare workers in one of the rural blocks of Jammu and Kashmir in North India about PCC. 

In our study, the majority of healthcare workers (95.6%) were females. This predominance of female participants reflects the composition of the healthcare workforce in India, where ASHA workers are almost exclusively female, and the nursing workforce is largely female-dominated [18]. All the participants were aged >30 years, possibly reflecting the experienced and established nature of the primary healthcare workforce in the study area. However, this may have influenced KAP findings and limited generalizability to males and younger healthcare workers.

In this study, we found that 59.1% of the healthcare workers possessed inadequate knowledge. It was in accordance with many studies across the world. Kassa et al., in their study from Ethiopia, revealed that only 31% of healthcare providers exhibited acceptable knowledge about PCC [13]. Another study from Ethiopia by Abayneh et al. demonstrated poor PCC knowledge by more than half of obstetric care providers [14]. A cross-sectional study involving nursing students from Sudan demonstrated that fewer than half (46.1%) possess adequate knowledge [15]. Similarly, a qualitative study by Ojukwu et al. suggested that general practitioners in the UK have a low understanding of the PCC recommendations [16]. Knowledge of the various vital components of PCC has also been identified as inadequate in our study. Also, a study from Malawi showed that 95% of health workers do not know about the details of PCC [17].

Preconception folic acid prevents neural tube defects in the fetus and also diminishes the likelihood of pre-eclampsia, abortions, low birth weight, small for gestational age, stillbirth, neonatal death, and autism in children [19]. Studies show that about one quarter of the women of reproductive age in India have folic acid deficiency [20]. In our study, 71.3% of healthcare workers recognized that folic acid should be initiated preconceptionally; however, only 36.5% were aware of the appropriate dosage and schedule. The implementation of this knowledge is even more inadequate, with merely 10.4% always prescribing preconception folic acid. Similarly, in a study conducted by Demilew et al., less than half (47.7%) of health professionals possessed sufficient knowledge, and only 9.7% had prescribed folic acid to women during the periconception period [21]. Research indicates that micronutrient supplementation started in pregnancy can correct important maternal nutrient deficiencies, but it is not sufficient to fundamentally improve child health. The preconception period provides a window of opportunity for interventions to improve health across generations [19]. This understanding was lacking among healthcare workers in our study, and 63.5% of them considered that it is sufficient to treat anemia just during pregnancy. It may be because, traditionally, antenatal care takes precedence over PCC. Also, in existing maternal healthcare services, frontline healthcare workers are more frequently engaged in antenatal registration, immunization, institutional delivery promotion, and postnatal services, which are well-established components of maternal and child health programs. In contrast, PCC remains a relatively underemphasized and evolving area within primary healthcare.

Obesity leads to adverse pregnancy outcomes: gestational diabetes, large-for-gestational age, pre-eclampsia, low Apgar score, infant mortality, severe maternal morbidity, and preterm birth. Weight management before conception mitigates these detrimental consequences. In India, the prevalence of obesity among pregnant women ranges from 12% to over 40% across different regions [3]. Our study indicates that 61.8% of healthcare workers were unaware of the unfavorable impact of obesity on pregnancy. Only one-fourth of them were engaged in dietary and weight management counseling. This aligns with a study by Waring et al., which indicates that one-third (29%) of obese women receive weight management counseling during preconception visits [22]. Another study by Kizirian et al. found that most of the general practitioners do not consider obesity a significant preconception issue [23].

IPV has a negative impact on the mother, child, and family. It is associated with delayed or absent prenatal care, depression, substance use during pregnancy, and low birth weight. Health care providers should be able to identify women with IPV and provide appropriate care. Polomon et al. reviewed 12 studies and found that the healthcare providers lack knowledge about IPV and are not prepared to deal with it as a health issue [24]. In our study, 66.1% of healthcare workers were also unaware that IPV can adversely affect the health of women and mothers. This may be attributed to limited formal training or sensitization regarding the identification, screening, and counseling of women experiencing IPV. In addition, sociocultural stigma surrounding IPV may discourage open discussion on this topic in healthcare settings, leading to underrecognition of its health consequences.

The understanding of mental health, rubella vaccination, and sexually transmitted diseases (STDs) was commendable in our study. This may be attributed to the longstanding National Programmes in the country, such as the Universal Immunisation Programme since 1985, the National Mental Health Programme since 1982, and the National STDs and AIDS Control Programmes. It highlights that a comprehensive program designed specifically for PCC like these programs, may enhance the PCC awareness and delivery.

In our study, the attitude of healthcare workers towards PCC is poor, and 61.7% of them were of the view that it was outside the scope of their services. It may be because their focus is on pregnancy care and not pre-pregnancy care. Additionally, heavy workload and time constraints may contribute to the perception that PCC is an additional responsibility beyond their existing duties. A systematic review by Goosens et al., encompassing 31 research articles, indicates that healthcare workers lack clarity regarding their responsibilities in providing PCC, which is a significant impediment to its implementation [25].

The insufficient knowledge and adverse attitudes of healthcare workers reflected upon the practice of PCC in our study, with only 18.3% demonstrating good self-reported practice. Other studies have found similar results, with only 13% of Australian primary healthcare nurses routinely engaging in PCC [26]. A study from Ethiopia indicates that the practice of PCC among healthcare providers is also inadequate, at 34.5% [14].

India has around 16 policies and 10 national programs that deliver different components of PCC in a fragmented fashion [27]. The effective implementation of PCC necessitates the establishment of national policies, the development of comprehensive PCC packages, and their integration into existing health care programs within current health care systems [12,28,29]. In our study, 59.9% believed that they could readily incorporate the elements of PCC in their daily practice for all eligible individuals.

In the present study, the KAPs of PCC were significantly associated with the educational level and professional background of the healthcare workers. This may be attributed to enhanced learning opportunities associated with higher levels of education and professional advancement. Medical officers and health workers possessing diplomas, such as ANM, GNM, MPHW, and LHV, have some reference to PCC in their curriculum. ASHA personnel are lacking due to the absence of a PCC-specific training program. A study conducted by Abayneh et al. also demonstrated that the probability of possessing PCC knowledge was three times greater among degree holders compared to diploma holders [14]. A study by Desale, too, found that the practice of PCC is substantially correlated with educational status, profession, and practice setting [30]. It underscores the importance of including PCC in the curriculum of healthcare professionals and regular training programs. Chutke et al. also highlighted the necessity for PCC guidelines and training courses for capacity building and the significance of coordination and cooperation among different levels of health workers at the community level [28].

The possible contributors to the poor KAP regarding PCC among healthcare workers may be insufficient training, lack of formal PCC protocols at the primary healthcare level, role ambiguity across different cadres of healthcare providers, heavy workload due to involvement in multiple existing health programs.

Furthermore, in the Indian public healthcare system, there is currently no distinct or dedicated component for PCC under existing national health programs. These programs are predominantly pregnancy-centered rather than pre-pregnancy-centered, resulting in comparatively less emphasis on preventive interventions before conception

Strengths of the study

The study addresses an important and underexplored public health issue and provides useful baseline data on the KAP of PCC among rural healthcare workers of Jammu and Kashmir. The inclusion of multiple cadres of healthcare providers and the use of structured data collection are its notable strengths. 

Limitations of the study

The present study has certain limitations. Being a cross-sectional study, causal relationships between variables could not be established. The study was conducted among all healthcare providers from the public sector within a single block, with no representation from urban or private healthcare settings, which may limit the generalizability of the findings.

Although the questionnaire underwent expert validation, translation-back translation, and pilot testing, internal consistency reliability measures such as Cronbach’s alpha were not formally assessed, which may be considered a limitation.

Furthermore, since the assessment of different domains was done by a self-administered questionnaire, the study population was susceptible to recall bias and social desirability bias. As the practice-related responses were self-reported for the preceding six months, the actual clinical practice of PCC by healthcare workers could not be independently verified. Another limitation of the study was that only bivariate analysis was performed. Due to the relatively small sample size and the limited number of participants in some categories, multivariable analysis could not be carried out reliably. As a result, the observed associations may have been influenced by other factors such as age, profession, work experience, or prior training exposure.

## Conclusions

The study revealed that the rural healthcare workers in Jammu and Kashmir, North India, had poor knowledge and a negative attitude towards PCC. They were practicing PCC in a very limited way.

Conducting well-structured training for healthcare workers may improve their knowledge and cultivate a positive attitude toward PCC, thereby enhancing its practice. In addition, the development of well-defined PCC packages and integrating them with existing maternal and reproductive health programs, particularly antenatal care, could potentially improve the delivery of PCC. Emphasizing PCC at the primary health care level can fill the gap in the continuum of care, leading to improved health outcomes for women, children, and future generations. Further studies are required to understand the barriers to the implementation of PCC and to formulate context-specific policies regarding PCC.

## Figures and Tables

**Figure 4:**
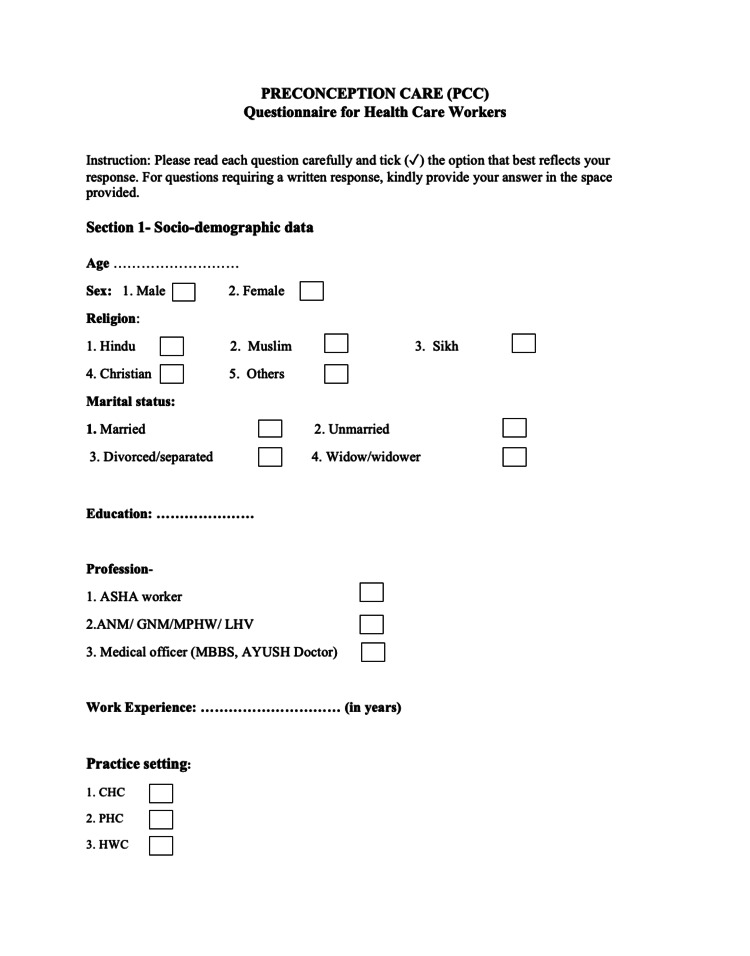
KAP questionnaire: section 1 - sociodemographic data

**Figure 5:**
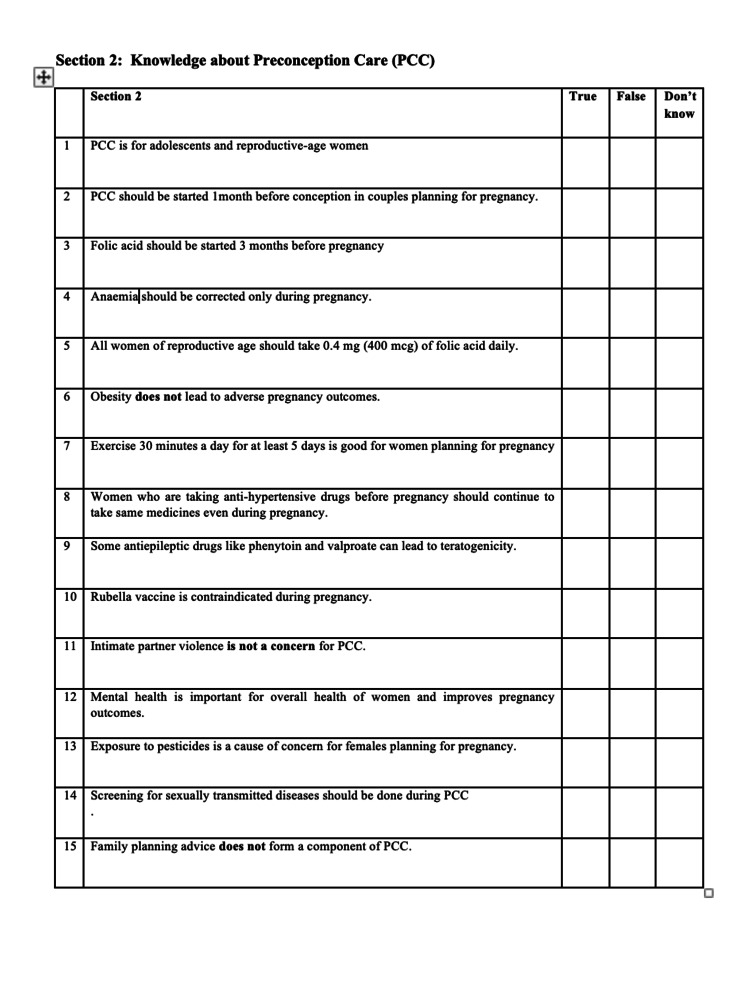
KAP questionnaire: section 2 - knowledge about preconception care

**Figure 6:**
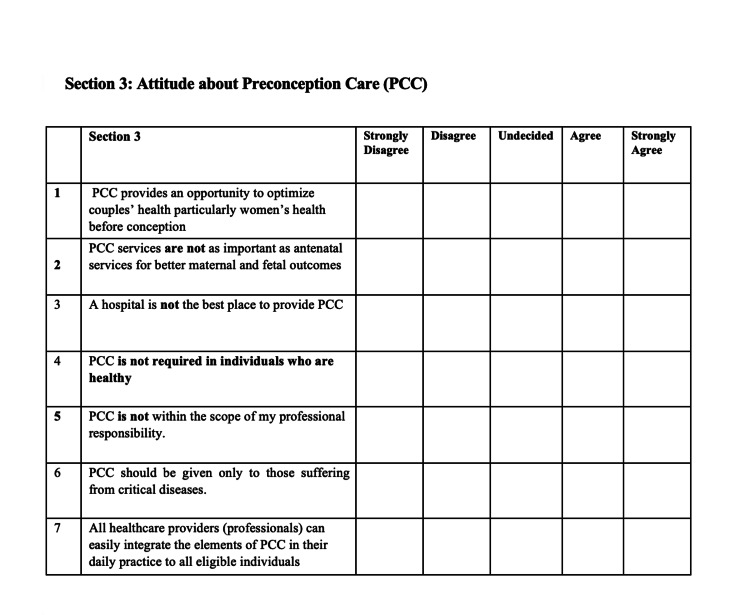
KAP questionnaire: section 3 - attitude about preconception care

**Figure 7:**
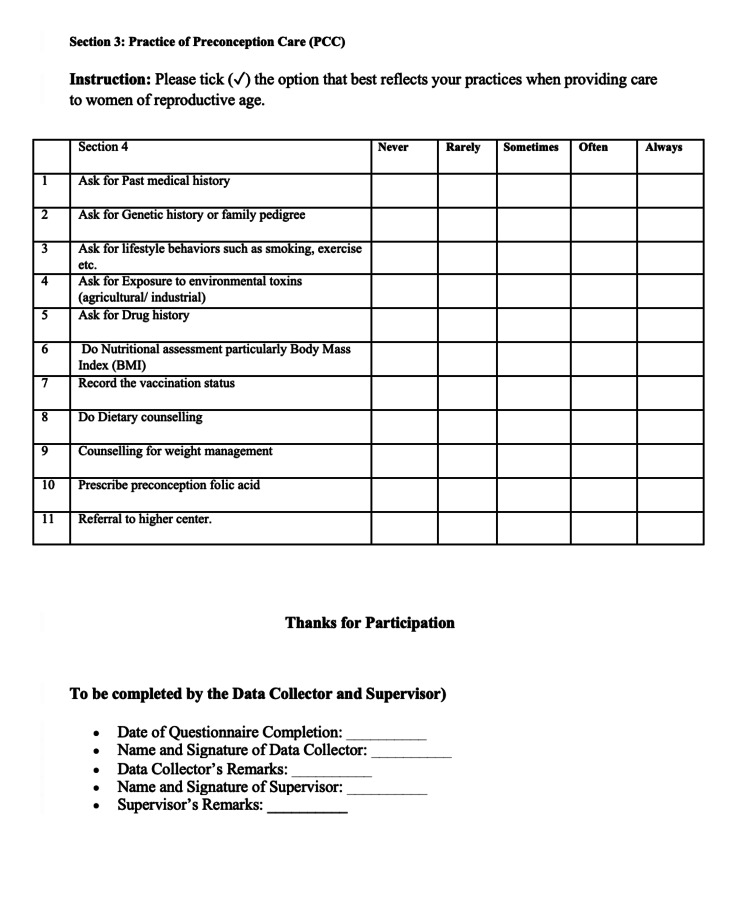
Practice of preconception care
